# Expansive open-door laminoplasty secured with titanium miniplates is a good surgical method for multiple-level cervical stenosis

**DOI:** 10.1186/s13018-014-0049-8

**Published:** 2014-08-21

**Authors:** Kuang-Ting Yeh, Tzai-Chiu Yu, Ing-Ho Chen, Cheng-Huan Peng, Kuan-Lin Liu, Ru-Ping Lee, Wen-Tien Wu

**Affiliations:** 1 Institute of Medical Sciences, Tzu Chi University, No. 701, Zhongyang Rd., Sec. 3, Hualien 97004, Taiwan; 2 Department of Orthopedics, Hualien Tzu Chi Hospital, Buddhist Tzu Chi Medical Foundation, Hualien 970, Taiwan; 3 School of Medicine, Tzu Chi University, Hualien 97004, Taiwan

**Keywords:** Open-door laminoplasty, Cervical spondylotic myelopathy (CSM), Titanium miniplate

## Abstract

**Background:**

Laminoplasty is an effective procedure for treating cervical spondylotic myelopathy (CSM). Little information is available regarding the surgical outcomes of expansive open-door laminoplasty (EOLP) when securing with titanium miniplates without bone grafting. This study is aimed to elucidate the efficacy of and problems associated with EOLP secured with titanium miniplates without bone grafting, thereby enhancing future surgical outcomes.

**Methods:**

This is a retrospective study. The study participants comprised 104 patients who underwent cervical EOLP secured with titanium miniplates without bone graft for CSM treatment between August 2005 and March 2011. The clinical results were evaluated based on the Japanese Orthopedic Association (JOA) and Nurick scores. The radiographic outcomes were determined based on plain film and magnetic resonance imaging findings, which were assessed and compared.

**Results:**

Lateral cervical spine X-rays exhibited improvement in the Pavlov ratio of the spinal canal at 1 day postoperation, and this ratio did not change at 1 year postoperation. The mean cervical curvature from C2 to C7 decreased 0.21° ± 10.09° and the mean cervical range of motion was deteriorated by 35% at 12 months (*P* < 0.05). The Nurick score improved from 3.19 ± 1.06 to 0.92 ± 1.32 (*P* < 0.05). The mean JOA recovery rate was 75% ± 21.1% at 1 year. The mean level of postoperative neck pain at 3 months was 3.09 ± 2.31, as determined using the visual analogue scale (VAS). Increased age, concomitant thoracolumbar stenosis, depression disorder, and preexisting myelomalacia negatively affected the JOA recovery rate (*P* < 0.05). A decreased preoperative Nurick score and superior sensory function in the upper extremities were powerful predictors of an enhanced JOA recovery rate. The postoperative complications involved hematoma formation 0.9%, reversible C5 nerve palsy 2.8%, and moderate to severe neck pain (VAS ≥ 4) 42%. No cases of lamina closure or collapse were observed.

**Conclusion:**

EOLP secured with titanium miniplates without bone grafting is a safe and effective surgical method for treating most patients with CSM.

## Background and introduction

Cervical spondylosis is an age-related degenerative change in the spine. Radiographic evidence of cervical spondylosis can be observed in more than 85% of those greater than 60 years old [1]. In cervical spondylosis, herniated discs; osteophytes; arthritic facet joints; buckled, thickened, or ossified ligamentum flavum; and hypertrophy or ossification of the posterior longitudinal ligament may all cause multilevel cervical stenosis, resulting in spinal cord compression. Chronic compression of the cervical spinal cord causes the clinical syndrome of cervical spondylotic myelopathy (CSM) [2],[3]. In certain patients who exhibit developmental stenosis of the cervical spine, myelopathy may occur early in life, particularly after hyperextension injuries [4].

Patients who experience progressive, long-standing, or severe myelopathy are candidates for surgical decompression of the spinal cord [2],[5]–[7]. The options decompressing multilevel stenosis involve anterior or posterior approaches. The factors influencing the operative approach are the location of the cord compression, number of levels involved, sagittal alignment, instability, associated axial neck pain, and risk factors for pseudarthrosis [7],[8]. Anterior cervical discectomy with fusion (ACDF), anterior cervical corpectomy with fusion (ACCF), and a combination of both are major anterior approaches that directly eliminate the anterior compression. The posterior options are laminectomy without fusion, laminectomy with instrumented fusion, and laminoplasty. The posterior approach relies on the decompression by both the direct removal of offending posterior structures and indirect posterior translation of the spinal cord; thus, patients should undergo maintenance of lordosis or correctable kyphosis to permit adequate indirect decompression [5]. Laminoplasty is superior to laminectomy without fusion because it decreases perineural adhesion and late kyphosis. Compared with ACDF, ACCF, or laminectomy involving instrumented fusion, laminoplasty preserves motion segments and prevents fusion-related complications, including bone graft dislodgement, pseudarthrosis, and adjacent segment disease [5],[9].

Several types of laminoplasty exist such as Z plasty, open-door laminoplasty, and French-door laminoplasty with variable modifications [7],[10]. Laminar Z plasty was devised by Hattori in 1971, reported by Oyama in 1973 [11], and subsequently modified by Tomimura and Watanabe in 1984 and 1987, respectively. Because of their complexity, these techniques were not popular [9].

Expansive open-door laminoplasty (EOLP) was developed by Hirabayashi in 1977; which was fixed with suture material between a hinge-side facet capsule and opened laminae. French-door laminoplasty was first documented by Kurokawa in 1982. Both EOLP and French-door laminoplasty became popular, and multiple subtypes of these operations were developed [9]. Biomechanical studies have demonstrated that the range of motion (ROM) and stability of the cervical spine did not change immediately after expansive laminoplasty [12],[13]; however, in one study, the ROM significantly decreased after 6 months. Herkowitz determined that EOLP yields a decompression effect equivalent to that of laminectomy or anterior decompression with fusion [14],[15]. Moreover, previous reports on long-term surgical outcomes have indicated the usefulness of conducting laminoplasty to treat patients with CSM [16]. However, complications have been reported shortly after surgery, and 30%–60% of patients exhibited postoperative neck pain [17],[18], 0%–30% (4.7%) exhibited C5 palsy [19],[20] and 34% exhibited lamina closure [21],[22], thereby compromising the surgical results. In 1996, O'Brien et al. reported a method involving maxillofacial miniplates and screws for securing the laminae in their opened positions [23]. This design provides primary resistance against closure of the laminae. Conducting EOLP by using miniplates and screw fixation remains a popular technique for treating multilevel CSM; however, few clinical reports have been conducted regarding this method.

Various methods can be employed to open the laminae and fix them to prevent reclosure. When conducting EOLP, we use the scalp clip applier as a special laminae opening tool and fix the opened laminae by using self-bent titanium miniplates. In this study, we present the surgical outcome of treating 104 patients with multilevel CSM by using EOLP secured with titanium miniplates without bone grafting.

## Materials and methods

### Study population

This study involved a retrospective design. The participants comprised 104 consecutive patients who underwent EOLP secured with titanium miniplates to treat CSM between August 2005 and March 2011. Clinical diagnoses were made by conducting physical examinations and using plain radiography and magnetic resonance imaging (MRI). All patients who met the following criteria were included in the study: (1) bilateral hand clumsiness and an unsteady gait; (2) positive myelopathic signs and symptoms, such as increased tendon reflexes in the extremities, clumsiness in the hands, and impaired toe-to-heel tandem gait; and (3) C3–C7 stenosis without segmental instability or kyphosis. Those who exhibited a history of cervical spinal surgery or presented with myelopathy because of spinal cord tumors or injuries were excluded. Demographic data and comorbidities were collected and recorded.

**Figure 1:**
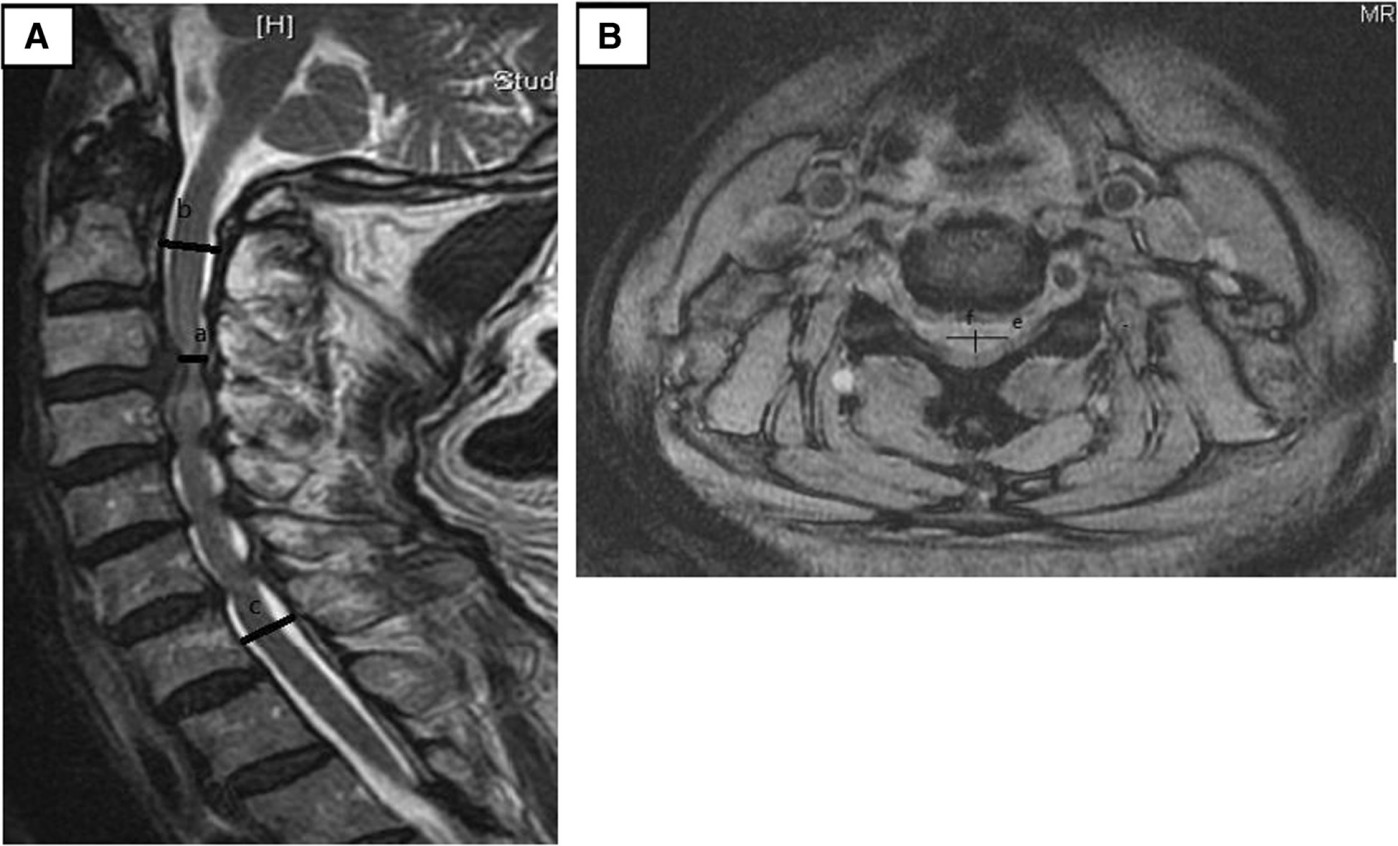
**T2 MRI image of cervical spine. (A)** Sagittal view. Sagittal compressive ratio = *a* × 2/(*b* + *c*). **(B)** Axial view. Axial compressive ratio = *f*/*e*.

The severity of myelopathy was rated using Japanese Orthopedic Association (JOA) [24],[25] and Nurick scores [26]. The surgical outcomes were further evaluated using the JOA recovery rate [24],[25]. The cervical curvature, ROM, and Pavlov ratio [27] (i.e., the canal-body ratio) were determined based on the plain films and the compressive ratios with or without preoperative myelomalacia were determined based on MRI (Figure 1); these factors all affected the postoperative outcomes [28],[29]. Patient conditions (e.g., age and gender) and comorbidities, such as diabetes mellitus (DM) and concomitant thoracolumbar (TL) stenosis, could also influence the evaluations of the surgical results [30]. Computed tomography (CT) scans and MRI studies were used to observe the stability at fixation sites and the postoperative decompression of the spinal cord.

We determined the Pavlov ratio at the C5 level based on neutral lateral X-rays. The cervical curvature was determined based on the lordotic angle, which was measured between the lower end plate of C2 and upper end plate of C7 in neutral lateral X-rays. The cervical ROM was defined as the difference in lordotic angle; from the lower end plate of C2 to the upper end plate of C7 between the extension and flexion lateral X-rays. The sagittal and axial compressive ratios from the preoperative MRI studies were estimated and recorded. This study was approved by the Research Ethics Committee of Hualien Tzu Chi Hospital, Buddhist Tzu Chi Medical Foundation (IRB101-100).

### Surgical procedures

The technique used in this study was modified from techniques that have been used in previous studies [23],[31] and is detailed as follows. The posterior approach was made from the C2 to T1 levels by using a midline longitudinal incision. The paraspinal muscles were subperiosteally dissected from the spinous processes to the lateral masses. The muscular insertions at the spinous process of C2 were protected from division. The intervals between the junction of the lamina and the facet joints were bilaterally developed from C3 to C7. On the hinge side, the outer cortex was removed using a 2-mm high-speed cutting burr to make a trough.

**Figure 2:**
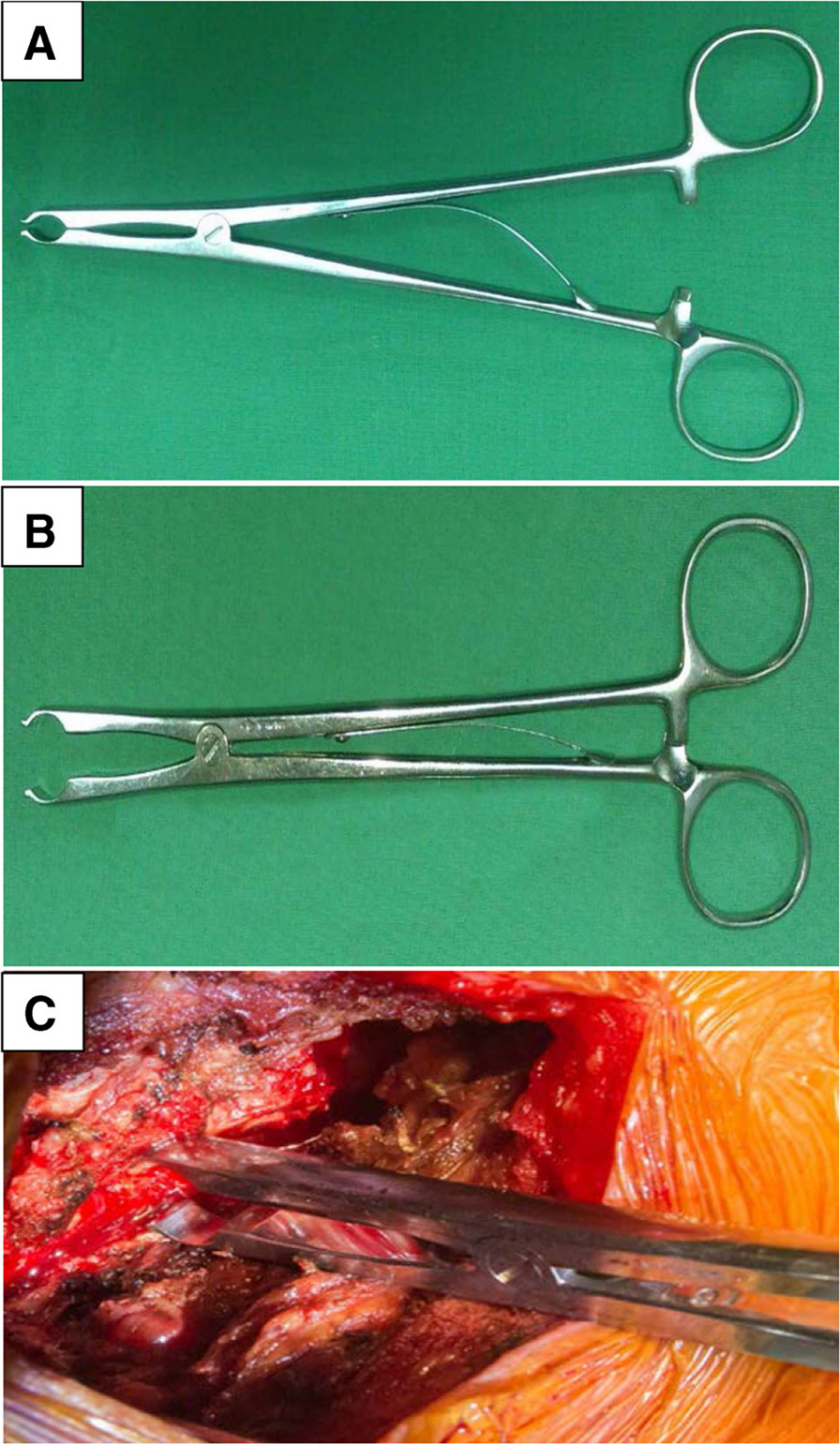
**Scalp clip applier (A and B) used as a spreader (C) for opening burred gutters of laminae.**

**Figure 3:**
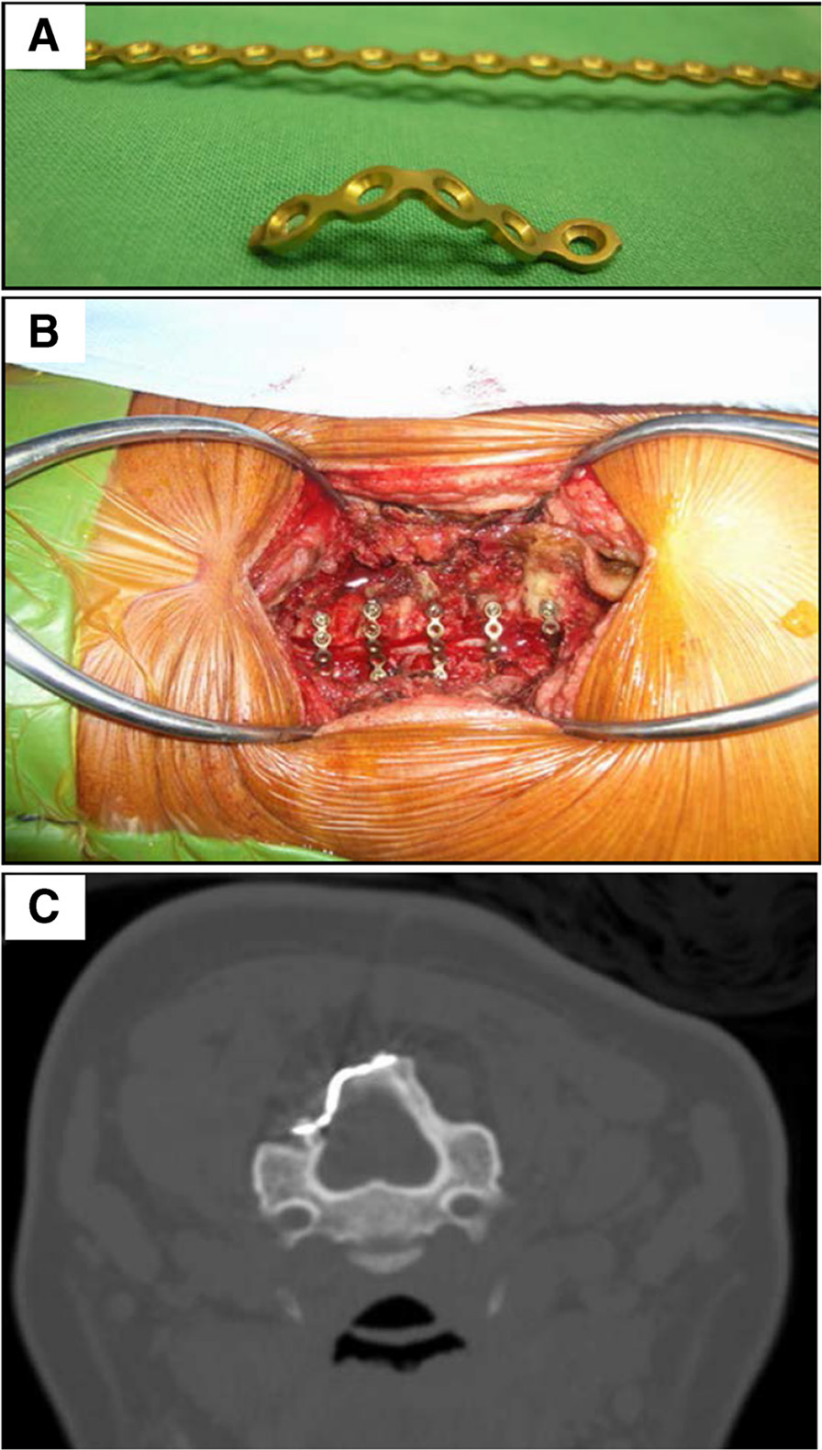
**Applying titanium miniplates. (A)** The plate was cut into pieces and they were bent into a wide-angled Z shape. **(B)** The open side was secured with five pieces of bent miniplates. The spinal cord was decompressed and inflated. **(C)** Each titanium miniplate was bent and applied to both the elevated laminae and lateral mass by fixing one miniscrew at each side.

On the open side, the 3-mm high-speed cutting burr was used to clean the outer cortex and cancellous bone. The remaining inner cortex was removed using a 1- or 1.5-mm Kerrison punch. The facet joints on both sides were protected from violation. The ligamentum flavum was divided between the C2 and C3 and C7 and T1 vertebrae. The laminae from C3 to C7 were then opened by a scalp clip applier (Mizuho Ika, Tokyo, Japan) as a spreader, followed by sequentially dividing the underlying ligamentum flavum on the opening side (Figure 2). Each lamina was maintained open and fixed with a 5-hole miniplate that was cut from a long, straight miniplate (Synthes 2.0 mm titanium miniplate 20 holes, adaption, Switzerland) and bent into the shape of wide-angled Z to fit both the cut edge of lamina and the lateral mass (Figure 3A). The well-bent miniplates were securely fixed with one miniscrew at each site at the C3 to C7 levels (Figure 3B,C). A rigid cervical collar was subsequently required for 3 months, and the patients were taught to perform neck extension exercises while protected by the collar.

### Outcome evaluation

#### Radiographic data

**Figure 4:**
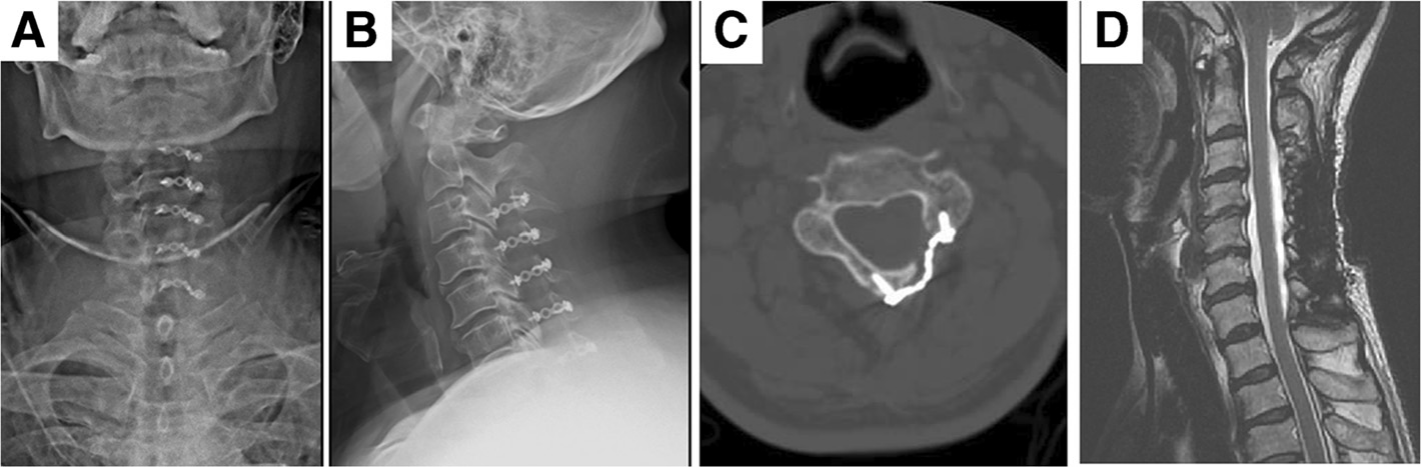
**Postoperative radiographic follow-up. (A)** AP view and **(B)** lateral view at postoperative 3 months. **(C)** CT scan at postoperative 6 months revealed well-positioned miniplate and screws and bone healing of hinge side. **(D)** MRI study at postoperative 12 months showed patent spinal cord without compression.

Neutral lateral cervical X-rays were captured preoperatively and at Day 0, 6 weeks, 3 months, 6 months, 1 year, and annually postoperation (Figure 4A,B). Dynamic X-rays were captured preoperatively and at 3 months, 6 months, and 1 year postoperation. CT scans were performed at 6 months postoperation and used to measure the union on the hinge side; a strong union was defined as cortical continuity at all axial cuts of the opened levels. MRI screenings were arranged at 12 months postoperation to evaluate the spinal cord decompression conditions. The cervical curvature, ROM, Pavlov ratio at the C5 level, and sagittal and axial compressive ratios were independently measured by two orthopedic doctors.

#### Clinical data

The neurological status was evaluated using the JOA [24],[25] and Nurick scores [26] preoperatively and at 12 months postoperation. Postoperative neck pain was evaluated using a 10-point visual analogue scale (VAS) at 2 weeks and 3 months postoperation. The JOA recovery rate at postoperative 12 months, which represents the degree of normalization after surgery, was calculated using the Hirabayashi formula: (Postoperative score − Preoperative score) × 100/(17 − Preoperative score) [24],[25]. We also recorded any postoperative complications.

### Statistical analysis

Data were presented as means ± standard deviation (SD). A paired *t* test was conducted to statistically analyze the difference between the preoperative and postoperative scores. A stepwise regression analysis was used to correlate the preoperative condition to the JOA recovery rate. A *P* value of <0.05 was considered statistically significant.

## Results

The participants comprised 67 men and 37 women, whose ages were 27–90 years old (mean = 60.13 ± 11.89 years old). The mean myelopathic symptom duration period was 14.86 ± 6.46 months. The mean period of symptom aggravation was 2.82 ± 1.1 months. All procedures were performed by one surgeon. The mean operation time was 122 min, and the mean blood loss was 245 ml. The mean postoperative follow-up period was 25.2 ± 3.2 months. Because no significant differences were observed between the surgical outcomes at 12 and 24 months postoperation, only the data until month 12 are presented herein.

### Radiologic evaluation

No hardware failure was observed during the follow-up period. The mean cervical curvature values before the operation and at 3 months postoperation were 9.7° ± 8.5° and 7.0° ± 9.7°, respectively (Table 1). The lordotic angle was restored to 9.5° ± 18.9° (*P* < 0.05) at 12 months postoperation. The mean Pavlov ratios (i.e., canal-body ratio) at the C5 level measured from X-rays of the lateral cervical spine were 0.66 ± 0.05 before the operation, 1.13 ± 0.10 immediately following the operation, and 1.12 ± 0.10 at 12 months postoperation, indicating significant improvement immediately following the operation (*P* < 0.05) and no collapse or laminae closure at 12 months postoperation (Table 1). The mean ROM values were 24.6 ± 12.2 degrees before the operation, 14.4° ± 7.2° at 3 months postoperation, and 15.99° ± 7.49° at 1 year postoperation (Table 1). The loss of ROM was approximately 35% at 1 year postoperation (*P* < 0.05). CT scans at 6 months postoperation demonstrated satisfactory stability levels and bony fusion over the hinge sides (Figure 4C). The preoperative mean sagittal compressive ratio and axial compressive ratio were 0.56 ± 0.06 and 0.36 ± 0.03, respectively. Preoperative myelomalacia was observed in 39 patients (37.5%), and significantly and negatively affected the JOA recovery rate (*P* < 0.05, Table 2). MRI screening at 1 year postoperation revealed patent spinal cord and substantial enlargement of the spinal canal (Figure 4D).

**Table 1:** **Preoperative/Postoperative radiographic status and their correlation to recovery rate (*N*** **= 104)**

**Items**	**Curvature**	**Pavlov ratio**	**ROM**
	**(Cobb angle)**		
Preop	9.7 ± 8.5	0.66 ± 0.06	24.6 ± 12.2
POD 1D	-	1.13 ± 0.10	-
POD 3M	7.1 ± 9.7	-	14.4 ± 7.2
POD 12M	9.5 ± 18.2	1.12 ± 0.10	16.0 ± 7.5
POD 1D-Preop^a^	-	−112.44*	-
POD 3M-Preop^a^	2.70*	-	15.62*
POD 12M-Preop^a^	0.12	−112.97*	14.57*
Preop vs. RR^b^	−0.10	0.04	0.06*

^a^*r* value; ^b^*t* value; *P < 0.05.

**Table 2:** **The correlation between preoperative condition and JOA score (*N*** **= 104)**

**Items**	**Sample size** ***n*** **(%)**	**Upper extremities motor function**	**Lower extremities motor function**	**Upper extremities sensory function**	**Lower extremities sensory function**	**Trunk sensory function**	**Bladder function**	**JOA score**	**Recovery rate**
Sagittal compressive ratio^a^	0.56 ± 0.06	0.06	−0.01	0.01	−0.04	−0.05	−0.14	−0.06	0.05
AP compressive ratio^a^	0.36 ± 0.03	0.06	−0.01	0.01	−0.04	−0.05	−0.14	−0.06	0.05
Myelomalacia^b^		−0.62	0.67	−0.05	1.21	2.36*	−0.08	0.67	−2.10*
No	65(62.5)	1.49 ± 0.90	1.46 ± 0.96	0.88 ± 0.45	0.42 ± 0.49	0.09 ± 0.29	0.22 ± 0.62	4.60 ± 1.99	0.78 ± 0.18
Yes	39(37.5)	1.38 ± 0.74	1.59 ± 0.88	0.87 ± 0.41	0.54 ± 0.51	0.31 ± 0.52	0.21 ± 0.57	4.87 ± 2.02	0.69 ± 0.25

^a^*r* value; ^b^*t* value; *P < 0.05.

### Clinical evaluation

Increased age, concomitant TL stenosis, and depression disorder negatively affected the JOA recovery rate (*P* < 0.05, Table 3). The Nurick scores increased from 3.2 ± 1.0 to 0.9 ± 1.3, and the JOA scores increased from 10.3 ± 2.9 to 15.0 ± 2.1, attaining significant improvement (*P* < 0.05). The VAS for postoperative neck pain at 2 weeks and 3 months was 6.4 ± 1.2 and 2.3 ± 2.2, respectively (Table 4). The JOA recovery rate was 75% ± 21.1% among the study patients. The results of a regression analysis indicated that preoperative Nurick scores and the sensory function in the upper extremities section of the JOA score were strong predictors of the JOA recovery rate (Table 5).

**Table 3:** **Demographic and comorbidity data and their correlation with JOA score (*N*** **= 104)**

**Items**	**Sample size,** ***n***	**Upper extremities motor function**	**Lower extremities motor function**	**Upper extremities sensory function**	**Lower extremities sensory function**	**Trunk sensory function**	**Bladder function**	**JOA score**	**JOA Recovery rate**
Age^a^	60.13 ± 11.89	0.04	0.01	0.01	0.13	−0.11	0.11	0.10	−0.25*
Gender^b^		−0.55	0.62	0.17	0.43	−0.80	0.28	−0.51	−0.02
Male	67	1.42 ± 0.80	1.55 ± 0.87	0.88 ± 0.44	0.48 ± 0.50	0.15 ± 0.35	0.22 ± 0.62	4.63 ± 1.96	0.75 ± 0.21
Female	37	1.51 ± 0.93	1.43 ± 1.04	0.48 ± 0.50	0.43 ± 0.50	0.22 ± 0.47	0.19 ± 0.56	4.84 ± 2.07	0.75 ± 0.20
Smoke^b^		0.40	1.30	1.20	−0.51	0.27	−0.08	0.94	0.52
No	89	1.44 ± 0.86	1.46 ± 0.96	0.85 ± 0.44	0.47 ± 0.50	0.17 ± 0.40	0.21 ± 0.63	4.65 ± 2.10	0.74 ± 0.21
Yes	15	1.53 ± 0.74	1.80 ± 0.67	1.00 ± 0.37	0.40 ± 0.50	0.20 ± 0.41	0.20 ± 0.41	5.00 ± 1.13	0.77 ± 0.21
Depression^b^		−1.95	−0.65	−0.11	−0.17	−4.38*	−3.59*	−1.97	−2.73*
No	97	0.86 ± 0.69	1.53 ± 0.95	0.88 ± 0.44	0.46 ± 0.50	0.19 ± 0.42	0.23 ± 0.62	4.80 ± 2.00	0.76 ± 0.20
Yes	7	1.49 ± 0.84	1.29 ± 0.48	0.86 ± 0.37	0.43 ± 0.53	0.00 ± 0.00	0.00 ± 0.00	3.29 ± 1.25	0.54 ± 0.26
DM ^b^		1.10	0.07	−1.34	0.63	0.82	−1.49	0.27	−0.10
No	83	1.41 ± 0.88	1.51 ± 0.90	0.90 ± 0.43	0.45 ± 0.50	0.16 ± 0.36	0.24 ± 0.65	4.67 ± 2.00	0.75 ± 0.22
Yes	21	1.62 ± 0.66	1.52 ± 1.07	0.76 ± 0.43	0.52 ± 0.51	0.24 ± 0.53	0.10 ± 0.30	4.81 ± 2.01	0.74 ± 0.16
CAD^b^		1.29	1.59	−0.61	0.65	−0.57	0.44	1.17	1.10
No	81	1.40 ± 0.84	1.43 ± 0.93	0.89 ± 0.44	0.44 ± 0.50	0.19 ± 0.39	0.20 ± 0.57	4.58 ± 1.87	0.74 ± 0.21
Yes	23	1.65 ± 0.83	1.78 ± 0.90	0.83 ± 0.38	0.52 ± 0.51	0.13 ± 0.45	0.26 ± 0.68	5.13 ± 2.38	0.79 ± 0.20
Previous neck trauma^b^		−0.62	−0.18	1.28	−0.24	0.94	−0.10	−0.29	0.34
No	79	1.48 ± 0.90	1.52 ± 0.99	0.85 ± 0.45	0.47 ± 0.50	0.15 ± 0.39	0.22 ± 0.65	4.73 ± 2.15	0.74 ± 0.21
Yes	25	1.36 ± 0.63	1.48 ± 0.71	0.96 ± 0.35	0.44 ± 0.50	0.24 ± 0.43	0.20 ± 0.40	4.60 ± 1.38	0.76 ± 0.20
Concomitant T-L stenosis^b^		−0.94	0.12	−1.69	0.64	−1.19	0.95	−0.24	−2.55*
No	62	1.52 ± 0.78	1.50 ± 0.82	0.94 ± 0.40	0.44 ± 0.50	0.21 ± 0.44	0.16 ± 0.48	4.74 ± 1.95	0.79 ± 0.18
Yes	42	1.36 ± 0.93	1.52 ± 1.08	0.79 ± 0.47	0.50 ± 0.50	0.12 ± 0.32	0.29 ± 0.74	4.64 ± 2.07	0.69 ± 0.23

Age is shown as mean ± SD. ^a^*r* value; ^b^*t* value; * *P* < 0.05.

**Table 4:** **Preoperative/Postoperative function status and their correlation to recovery rate (*N*** **= 104)**

**Items**	**Preop**	**Postop**	**Postop-preop**	**Postop-preop**		**Preop vs. RR**	
	**Mean (SD)**	**Mean (SD)**	**Mean (SD)**	***t***	***P***	***r***	***P***
Nurick score	3.2 ± 1.1	0.9 ± 1.3	−2.3 ± 0.9	26.18	<0.001	−0.50	<0.001
VAS	5.1 ± 1.8	2.3 ± 2.2	−2.9 ± 2.4	12.38	<0.001	−0.41	<0.001
JOA score	10.3 ± 2.9	15.0 ± 2.1	4.7 ± 2.0	−24.04	<0.001	0.50	<0.001
JOA upper extremities motor function	2.1 ± 1.0	3.5 ± 0.8	1.5 ± 0.8	−17.50	<0.001	0.43	<0.001
JOA lower extremities motor function	1.9 ± 1.2	3.4 ± 0.9	1.5 ± 0.9	−16.47	<0.001	0.37	<0.001
JOA upper extremities sensory function	0.6 ± 0.6	1.4 ± 0.5	0.9 ± 0.4	−20.57	<0.001	0.45	<0.001
JOA lower extremities sensory function	1.2 ± 0.7	1.7 ± 0.5	0.5 ± 0.5	−9.40	<0.001	0.22	0.026
JOA trunk sensory function	1.8 ± 0.5	1.9 ± 0.2	0.2 ± 0.4	−4.36	<0.001	−0.03	0.784
JOA bladder function	2.7 ± 0.7	2.9 ± 0.2	0.2 ± 0.6	−3.58	0.001	0.11	0.267

RR, recovery rate.

**Table 5:** **The predictive power of preoperative condition to recovery rate (*N*** **= 104)**

**Items**	**R**	**R square**	**R square change**	**Sig. F change**
Nurick score	0.50	0.25	0.25	0.00
Upper extremity sensory function	0.62	0.38	0.13	0.00
T-L spondylosis	0.66	0.43	0.06	0.00
Depression	0.68	0.46	0.03	0.03
VAS	0.70	0.49	0.02	0.04

### Complications

No mortality occurred after operation. One patient experienced a dural tear that required lumbar drain shunting. One patient had a postoperative hematoma that required immediate reoperation. Three patients exhibited transient postoperative C5 nerve palsy (MMT score < 3), but were completely recovered at 3 months postoperation. Five exhibited poor wound healing that required a prolonged or second admission for reoperation and antibiotic treatment. No neurological deterioration was noted. At 3 months postoperation, 10 patients had developed kyphosis of the cervical spine according to follow-up X-rays. Moderate to severe neck pain and VAS scores ≥ 4 were noted in 42% of patients at 3 months postoperation; however, these patients exhibited strong functional recovery rates.

## Discussion

CSM was proven to result from the narrowing of the normal anteroposterior cervical spinal canal to a critical threshold [13]. The normal cervical aging process, congenital narrowing aggravated by acute trauma and bony malformations were identified as the primary causes of cervical stenosis that result in myelopathy [32]. The prognosis of untreated myelopathy was typically poor and conservative treatment was largely ineffective in ceasing the progression of neurological deterioration [15]. Operations have become a mainstream method of treating. Anterior approaches, (primarily ACDF, ACCF, or a combination of both) could be used to eliminate anterior compression directly and correct kyphotic deformity and instability. These approaches are reserved for pathology less than or equal to three motion segments. Regarding multilevel CSM in four or more motion segments, posterior procedures are the primary treatment options [5]. Laminectomy, an accepted decompressive procedure for treating multilevel CSM, has been reported to cause an increased incidence of complications, including perineural adhesion, instability, and late kyphosis involving neurological deterioration. Laminectomy with instrumented fusion provides stability and prevents late kyphosis; however, problems related to perineural adhesion and fusion have been reported [5],[33]. Laminoplasty, through various modifications and improvements, is considered advantageous for expanding the spinal canal and can preserve the posterior structure of the cervical spine in an effort to secure stability and prevent the formation of a postlaminectomy membrane. In addition, numerous studies have reported satisfactory surgical outcomes among patients receiving laminoplasty [34]. Hirabayashi described EOLP based on a modification from O'Brien, and the method of using titanium miniplates to secure the opening of spinal canal has remained a popular surgical procedure [28],[31]. Customized miniplates have been developed to fix the opened laminae; however, these are expensive and the adaptation can be limited by local anatomy. In this study, we conducted the operations by using self-bent miniplates cut from a long, straight titanium miniplate; using this method is both cheaper and more popular than using customized miniplates. The self-bent miniplates were advantageous because they could be readily applied and shaped and provided effective stability. In our case series, no laminae collapse or implant dislodgement was indicated in the follow-up plain films or CT scans.

In the study of Ratliff, cervical curvature was decreased by 35% and ROM was decreased by 50% after laminoplasty [35]. The loss of ROM was progressive and plateaued at 18 months postlaminoplasty [36]. In the biomechanical study of Puttlitz, neck extension-flexion ROM was decreased by approximately 25% at 6 months postlaminoplasty and no obvious kinetic differences were observed between EOLP and French-door laminoplasty [12]. The study suggested that decreased intervertebral motion should be expected and early rehabilitation therapy should be considered. In the current study, significant decreases in cervical curvature and ROM losses were observed at 3 months postoperation. The cervical curvature had almost returned to preoperative levels at 1 year postoperation. However, the ROM was only partially restored. The mean loss of cervical ROM was 35% at 2 years postoperation. In this study, the reason for the improved cervical curvature and ROM from 3 months to 1 year postoperation was aggressive rehabilitation after removal of the neck collar at 3 months postoperation. Lamina closure has been associated with unsatisfactory clinical outcomes after laminoplasty. It can be defined as a decrease of greater than 10% in the canal-body ratio (i.e., Pavlov ratio) [21]. In the study of Matsumoto, 34% of patients receiving open-door laminoplasty without plates or spacers on the open sides developed lamina closure [21]. In the current study, no cases of this complication were observed at 12 months postoperation. Our bent miniplates provided initial resistance and good stability for elevated laminae.

The surgical outcomes and risk factor analyses were primarily assessed using the JOA recovery rate; despite the popularity of this method, it involves certain limitations. Based on the preoperative JOA score, the actual surgical recovery could be distinct among patients who exhibit the same recovery rates. The recovery rate is low among patients who exhibit a low preoperative JOA score, even if they attain the same postoperative scores. Thus, we analyzed the six individual sections of the JOA score to determine how the levels of postoperative improvement and preoperative severity were correlated with the surgical outcome. We also used the Nurick score as a secondary method of assessing postoperative conditions. We determined that sensory and motor deficit in the limbs were highly correlated with the recovery rate, particularly the sensory parts of the upper extremities. We proved that the Nurick score was a strong predictor of surgical outcome. Furthermore, numerous factors affect the postoperative outcomes of patients with CSM. The prognostic factors of outcome after undergoing expansive laminoplasty to treat CSM have been identified as age [37], symptom duration, comorbidities [38], congenital narrow spinal canal [29], myelopathy severity, myelomalacia observed in MRI scans [11], and sagittal cervical alignment [15],[30]. Although each factor is essential for determining the prognosis of postoperative neurological improvement, no reports have described the factor most critical for predicting the surgical outcome. In the current study, increased age, depression disorder, and myelomalacia significantly and negatively affected the surgical outcome; however, further observation and follow-up is necessary.

In this study, the surgical outcomes of laminoplasty in patients with CSM were observed for an average of 25 months. No further deterioration was observed in the clinical and radiographical follow-up results after 12 months postoperation, indicating that surgical outcomes stabilize at month 12. Thus, only data until month 12 are presented in this study. No progression of cervical myelopathy was observed among the patients. Only three patients demonstrated evident C5 palsy (MMT score < 3), and this incidence (2.8%) was relatively low compared with that of the average values (4.7%) that have been reported in the literature [19],[20]. Substantial posterior shifts of the spinal cord caused by excessive expansion can readily cause the development of C5 palsy [20]. This problem could be solved by placing a medial trough as a hinge and lowering the lamina opening to minimize excessive posterior cord drift.

Five patients demonstrated poor wound healing and received prolonged or second admission to undergo debridement and antibiotic treatment. All five patients exhibited Type II DM, which yields elevated wound complication rates; thus, we consulted with metabolism doctors to control patient glucose levels by using medication and diet controls. No subsequent deep tissue infection or osteomyelitis was observed. To prevent severe complications, urgent debridement and antibiotic treatment followed by in-patient wound observation should be administered to those who exhibit poor wound healing. No cases of screw loosening occurred, and we carefully verified the screw purchasing quality intraoperatively. Thick screws are chosen or the fixing of original screws to the other bone area of the opened laminae is warranted when the screws tend to loosen.

Ten patients had developed kyphosis at 3 months postoperation. The reasons for the kyphotic change may be related to disrupted dorsal ligamentous structures, compromised extensor musculature, and the force from the less mobile segment; these factors may be related to late rehabilitation. Several modified surgical techniques have been developed to reduce postoperative neck pain after laminoplasty [39]. Early postoperative ROM exercises and a decreased period of neck collar protection may improve postoperative neck pain and decrease the loss of cervical ROM [40]. In the current study, the average level of moderate to severe postoperative neck pain was 42% at 3 months postoperation; this rate was similar to those reported in previous studies [17],[18]. The modifications that we implemented required decreased surgical dissection after the careful repair of the semispinalis cervicis muscle and nuchal ligament. In addition, the patients were provided early education regarding neck extension exercise to be practiced while wearing their protective neck collars. After March 2011, we implemented additional changes to our surgical procedures to minimize surgical trauma and reduce postoperative neck pain.

Based on the literature, the mean surgical time for various kinds of laminoplasty was approximately 50–230 min and the mean intraoperative blood loss was 52–370 ml [26],[41],[42]. To conduct the current procedure, we used a scalp clip applier to safely and gently open the laminae, subsequently fixing the opened laminae with self-bent titanium miniplates. The mean blood loss amount and average surgical times were within the normal ranges and the surgical outcomes were satisfactory. We suggest that surgical techniques be chosen based on the experience of the surgeon with specific procedures.

Regarding potential study limitations, the total follow-up period was short and no control group was formed. In addition, no patient-based outcome measures, such as the short-form health survey or subjective satisfaction measure, were assessed in this study. The current results demonstrated that conducting EOLP with miniplates is a safe and effective procedure; furthermore, it yields a low complication rate and can stop the progressive loss of or restore neurological function. The recovery rate in this study was more than 75%, which is comparable to that in previous reports [43],[44]. No patients in this study required revision to correct fixation failure, which could have caused lamina closure. This demonstrates that internal fixation facilitates maintaining the position of the lamina.

## Conclusion

EOLP secured with titanium miniplates without bone grafting appears to be a safe and effective method of arresting the progression of myelopathy; this method yields marked functional improvement in most patients with CSM.

## Competing interests

The authors declare that they have no competing interests.

## Authors' contributions

All authors had substantial contributions to the conception and design of the study and giving of the final approval to the manuscript. KTY, TCY, and WTW participated in the data acquisition. KTY and TCY were responsible for the data interpretation and writing of the manuscript. WTW and RPL were responsible for the manuscript modification and concept clarification. All authors have read and approved the final manuscript.
